# The Potential of *Zanthoxylum acanthopodium* DC. as Immunomodulators: Formulation, Activity Testing, and Extract Profiling

**DOI:** 10.3390/ph18071001

**Published:** 2025-07-03

**Authors:** Damaris Br. Hutapea, Yasmiwar Susilawati, Muhaimin Muhaimin, Riezki Amalia, Aisyah Tri Mulyani, Anis Yohana Chaerunisaa

**Affiliations:** 1 Doctoral Program in Pharmacy, Faculty of Pharmacy, Padjadjaran University, Bandung 45363, Indonesia; damaris21001@mail.unpad.ac.id; 2 Department of Biological Pharmacy, Faculty of Pharmacy, Padjadjaran University, Bandung 45363, Indonesia; yasmiwar@unpad.ac.id (Y.S.); muhaimin@unpad.ac.id (M.M.); 3 Department of Pharmacology and Clinical Pharmacy, Faculty of Pharmacy, Padjadjaran University, Bandung 45363, Indonesia; riezki.amalia@unpad.ac.id; 4 Magister Program in Pharmacy, Faculty of Pharmacy, Padjadjaran University, Bandung 45363, Indonesia; aisyah17015@mail.unpad.ac.id; 5 Department of Pharmaceutics and Pharmaceutical Technology, Faculty of Pharmacy, Padjadjaran University, Bandung 45363, Indonesia

**Keywords:** andaliman, immunomodulator, soft capsule, *Zanthoxylum acanthopodium* DC.

## Abstract

**Background/Objectives**: One of the plants found in Indonesian forests that has potential as an herbal medicine is andaliman (*Zanthoxylum acanthopodium* DC.). The fruit of *Z. acanthopodium* contains phenolic compounds that are known to modulate the immune response. The purpose of this study is to determine the extract profile and immunomodulatory activity of *Z. acanthopodium* fruit and to develop a soft capsule formulation of the extract in the form of emulsion, which stabilizes and acts as an immunomodulatory candidate. **Methods:** Extract profiling was conducted by liquid chromatography UHPLC–HRMS, and the predicted molecular structure was then used to search for the name of the compound using the mzcloud database. Immunomodulatory activity of the extract and its emulsion was assessed using a lymphocyte viability assay. The extract emulsion to be encapsulated as a soft capsule was developed by employing different types of oil and solubilizer in the oil phase, and a water phase containing the extract and two types of emulsifiers. **Results:** The chemical composition of andaliman extract was analyzed, including total phenolic content (4%), total flavonoid content (0.35%), and quercetin content (0.13%). Based on LC-HRMS analysis, eleven compounds derived from the ethanolic extract of andaliman were identified as potential immunomodulatory agents. The F3.3F formulation, which contains 30% MCT oil phase with solubilizer lauroyl-PEG-32 glycerides and a water phase with 35% Polysorbat (Tween) 80 emulsifier, provided the most stability. This stability is attributed to the presence of the Tween 80 emulsifier, which has superior wetting and washing functions, strong detergency, and good emulsifying properties compared to the PEG emulsifier used in formulation F3.3E. The survival rates in the lymphocyte cell viability test results indicate that treatment with andaliman extract (173.697% at 15.625 ppm; 174.923% at 31.25 ppm; 168.457% at 62.5 ppm) was better than treatment with kojic acid (144.375% at 15.625 ppm; 137.891% at 31.25 ppm; 146.345% at 62.5 ppm), used as the immunomodulatory agent standard. **Conclusions:** This study highlights the potential of andaliman extract as an immunomodulatory agent to be developed as an emulsion in a soft capsule.

## 1. Introduction

*Zanthoxylum acanthopodium* DC., commonly known as andaliman, is a plant native to Indonesian forests with significant potential as a medicinal agent. The fruit is frequently used as a culinary spice. This plant’s bark, roots, and leaves have traditionally been utilized to treat various ailments, including stomachaches, toothaches, coughs, rheumatism, and back pain. Preliminary research has identified the presence of several phytochemicals in andaliman, such as alkaloids, flavonoids, steroids, triterpenoids, and saponins [1]. Several studies have demonstrated a range of biological activities in this plant, including larvicidal, anti-inflammatory, analgesic, antimicrobial, antioxidant, and antifungal properties [2]. Species in the *Zanthoxylum* genus are known for their essential oils, which are rich in terpenoid compounds. The essential oil extracted from andaliman leaves has been shown to inhibit the growth of *Colletotrichum gloeosporioides* and *Botryodiplodia theobromae*, both pathogenic fungi in plants [3]. The antioxidant activity test of the ethanol extract of andaliman revealed an IC_50_ value of 84.1 ± 0.47 µg/mL, which is more potent than that of the n-hexane and ethyl acetate extracts [4]. Additionally, andaliman fruit extract has demonstrated the ability to scavenge singlet oxygen [5]. The essential oils contained in its fruit are believed to contribute to its antibacterial and antioxidant activities [6]. Furthermore, andaliman exhibits potential as an immunomodulator and has been associated with anticancer, antitumor, diaphoretic, antispasmodic, and vermifuge properties [7,8].

Most of the chemical constituents in plants (e.g., flavonoids, glycosides, and phenolics) are water-soluble (hydrophilic) compounds that dissolve in polar solvents. This characteristic affects their interaction with biological cells, primarily composed of lipid-based membranes. Consequently, these compounds often exhibit poor bioavailability as they cross the lipid-rich outer membrane of cells [9]. Poorly soluble drugs pose significant formulation challenges, particularly in achieving adequate absorption within lipid-soluble environments. One strategy to overcome this limitation is to develop a liquid formulation in which the drug is either dissolved or suspended in a lipophilic matrix system [10].

Soft capsules are dosage forms with a gelatin-based soft shell that encapsulates a liquid or semi-solid matrix. The drug compound incorporated into a soft capsule must be in the form of a solution or suspension, which is dispersed in a carrier that may be hydrophilic (e.g., polyethylene glycol), lipophilic (e.g., vegetable oil triglycerides), or a combination of both [10]. Active pharmaceutical ingredients in plant extracts can be developed into soft capsule formulations, particularly when the active compounds are poorly water-soluble. The extract can be processed into a microemulsion system (a preconcentrate) before encapsulation. A “preconcentrate” refers to the carrier system within the soft capsule, composed of a blend of lipophilic and hydrophilic liquids and surfactant. This emulsion system typically forms droplets with sizes in the micrometer range [10].

The microemulsion system in soft capsules offers significant advantages in dissolving the active drug. Microemulsion droplets (essentially surfactant micelles) enhance the diffusion of active drugs in gastrointestinal fluids, thereby improving their pharmacokinetic characteristics. This concept can be applied to natural extracts with low solubility, which facilitates better absorption and bioavailability. This study employed an emulsion-based formulation for andaliman extract in soft capsules as a carrier system. Several factors influencing this system include the type and concentration of excipients. Therefore, it is essential to investigate the effect of solvents and emulsifiers andaliman soft capsules formulation, as well as the stability study and immunomodulator activity [10].

The pharmacological effects of andaliman are primarily attributed to its secondary metabolites, particularly phenolic compounds, which have been reported to exhibit antioxidant, anti-inflammatory, and immunomodulatory properties [11,12]. In addition to phenolics, andaliman also contains alkaloids, flavonoids, steroids, triterpenoids, and saponins. Among these, flavonoids are well-known for their wide range of pharmacological activities, including immunomodulatory effects [13]. Thus, andaliman demonstrates strong potential for development as an immunomodulator due to two key groups of bioactive compounds—phenolics and flavonoids—that have been scientifically proven to have immunomodulatory activities [14].

The immune system is responsible for the body’s ability to combat pathogens by rejecting foreign substances entering the body, thereby preventing disease. Immunomodulators work by suppressing, stimulating, or modulating various immune system components—specific and nonspecific—and can be derived from natural or synthetic sources [15]. The immune system’s functionality can be assessed by measuring the viability of lymphocytes. These lymphocyte cells play a critical role in immune defense, as they are capable of secreting antibodies that specifically recognize and bind to molecules expressed by pathogenic cells, such as lipopolysaccharides (LPS) from gram-negative bacteria [16].

Various plants are known to stimulate components of the specific immune system (e.g., T cell proliferation and antibody-producing B cells) and the nonspecific immune system (e.g., macrophages and natural killer [NK] cells) [17]. Most plant-derived phenolic compounds influence nonspecific immune responses primarily by enhancing phagocytosis and promoting the proliferation of lymphocytes and neutrophils [18]. To evaluate the immunomodulatory effects of andaliman fruit, which is known to contain flavonoids and polyphenol compounds, this study conducted an in vitro immunomodulatory activity test using the ethanol extract of the fruit on lymphocyte cells. The immunomodulatory activity was assessed by calculating the percentage of cell viability in lymphocyte cultures, with and without lipopolysaccharide (LPS) induction, after exposure to various concentrations of the ethanol extract. The water-soluble tetrazolium (WST-8) assay was used for this evaluation. The extract was analyzed using liquid chromatography–high-resolution mass spectrometry (LC-HRMS) to identify the compound groups responsible for the observed immunomodulatory activity. Additionally, the extract was formulated into a soft capsule to enhance its solubility and bioavailability. The andaliman extract emulsion was developed by optimizing the type of emulsifier and the type and amount of oil used as its lipophilic matrix. The immunomodulatory activity was also conducted on the extract emulsion to ensure that the formulation process did not alter its biological activity.

## 2. Results and Discussion

### 2.1. Characterization of Standard Parameters of Andaliman Fruit

#### 2.1.1. FTIR-ATR Testing

**Figure 1:**
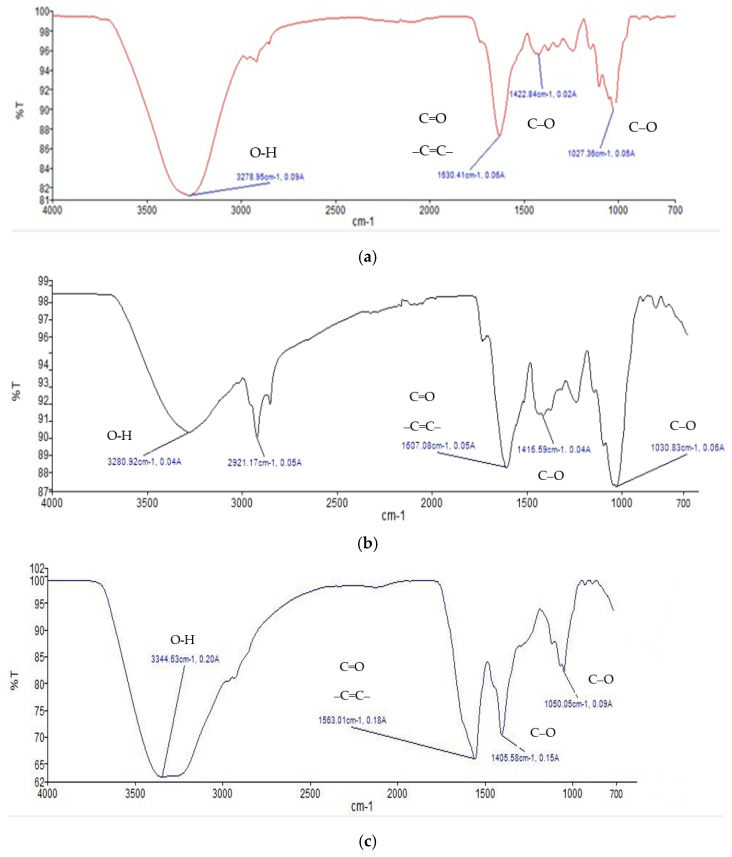
FTIR profile of (**a**) fresh andaliman fruit, (**b**) andaliman simplicia, and (**c**) ethanol extract of andaliman fruit.

Figure 1 shows FTIR profile of fresh andaliman fruit (a), andaliman simplicia (b), and ethanol extract of andaliman fruit (c). Meanwhile, Table 1 shows the interpretation of functional groups in the FTIR-ATR profile.

The FTIR testing results indicate that andaliman contains alcohol compounds (characterized by OH functional groups) at approximately 15.99% and carboxylic acid compounds (also with OH functional groups) at approximately 3.08%.

#### 2.1.2. Determination of Specific Parameters of Andaliman Fruit Extract

Determining specific parameters aims to provide an overview of the chemical compound content in the extract derived from simplicia, which is related to the reproducibility of its pharmacodynamic activity. This also includes the solubility of conventional medicinal ingredients in water or organic solvents. The solubility of natural compounds in organic solvents is a reference for the formulation of extract-based preparations. In contrast, solubility in water is helpful as a reference for traditional use by the community in the form of decoctions or infusions. The results of the specific and nonspecific parameter analysis of Andaliman fruit extract are shown in Table 2.

The measurement of specific gravity aims to provide an overview of the physicochemical properties of the extract, particularly in assessing the presence of contaminants based on changes in mass per unit volume [21]. Based on the results, the specific gravity of the andaliman fruit extract was found to be 0.81 g/mL, indicating a density of ≤1. This suggests that the sample can easily mix with water and vice versa. The drying loss percentage of andaliman fruit simplicia was 13.6 ± 0.4%. This value is higher than the measured water content, as the drying process may also cause the evaporation of other volatile compounds in addition to water. Determining total ash content provides insight into the amount of residue, physiological and non-physiological ash, remaining after incineration. This value reflects the quality, authenticity, and purity of the sample. A higher ash percentage generally indicates a better mineral content.

##### Metal Contamination Level

According to the Indonesian FDA Regulation No. 12/2014, the maximum allowable levels of heavy metal contamination are as follows: lead (Pb) ≤ 10 mg/kg, cadmium (Cd) ≤ 0.3 mg/kg, and mercury (Hg) ≤ 0.5 mg/kg. The results indicated that the heavy metal levels in andaliman fruit were well below the permissible limits, confirming that the sample was safe for use based on heavy metal contamination standards. Table 3 shows data on metal contamination levels in andaliman fruit extract.

#### 2.1.3. Phytochemical Screening

The results of the phytochemical screening of the ethanol extract of andaliman fruit revealed the presence of flavonoids, saponins, tannins, steroids, and coumarins [4]. Several studies have confirmed that these secondary metabolites contribute to the pharmacological properties of andaliman fruit, which has been shown to possess antibacterial [22,23,24,25], antioxidant [14], anticancer [26], anti-inflammatory [14,27], antidiabetic [28], anti-acne [22], anti-aging [22], antifertility [29], antifungal [24,30], and immunomodulatory activities [12,14].

#### 2.1.4. Thin Layer Chromatography (TLC) Profile of the Extract

**Figure 2:**
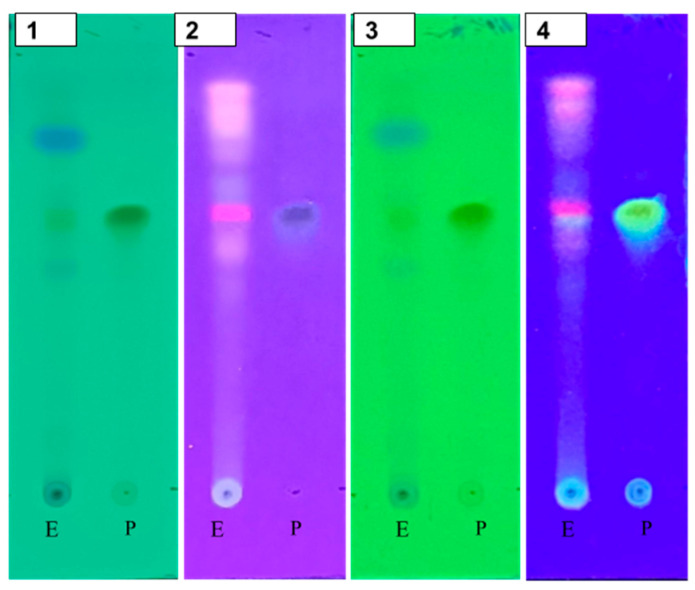
TLC profile of the 70% ethanol extract of andaliman fruit extract (E); quercetin (standard) (P). Visualization was performed under UV light at 254 nm (**1**), UV light at 366 nm (**2**), citroborate reagent under UV light at 254 nm (**3**), and citroborate reagent under UV light at 366 nm (**4**).

The thin layer chromatography (TLC) profile (Figure 2) was examined as a qualitative test to identify the presence of quercetin in the andaliman extract. The stationary phase used was a silica gel F_254_ TLC plate, and the mobile phase consisted of ethyl acetate/n-hexane/formic acid (7:3:0.5). The TLC plate was first observed under UV light at wavelengths of 254 nm and 366 nm. Subsequently, the plate was sprayed with a citroborate reagent, heated at 105 °C for 10 min and observed under UV light at 366 nm [31]. Spots on the TLC plate from the andaliman extract appeared parallel to the reference standard, with both displaying an Rf value of 0.62. The standard quercetin spot exhibited green fluorescence, which matched the extract. This similarity in Rf values and fluorescence indicated the presence of quercetin compounds in the extract. After spraying with citroborate and heating, the spots observed under UV 366 nm showed intensified yellow-green fluorescence, confirming the presence of flavonoids [32].

#### 2.1.5. Isolation of Quercetin

##### TLC Analysis and Isolation of Quercetin

The purity of the isolation results was proven by performing TLC using ethyl acetate/n-hexane/formic acid (7:3:0.5) eluents, observed under UV light. Based on the TLC results, it can be stated that the most appropriate eluent for isolating flavonoid compounds is ethyl acetate/n-hexane/formic acid (7:3:0.5) eluent, which shows good spot separation with an Rf of 0.62.

**Figure 3:**
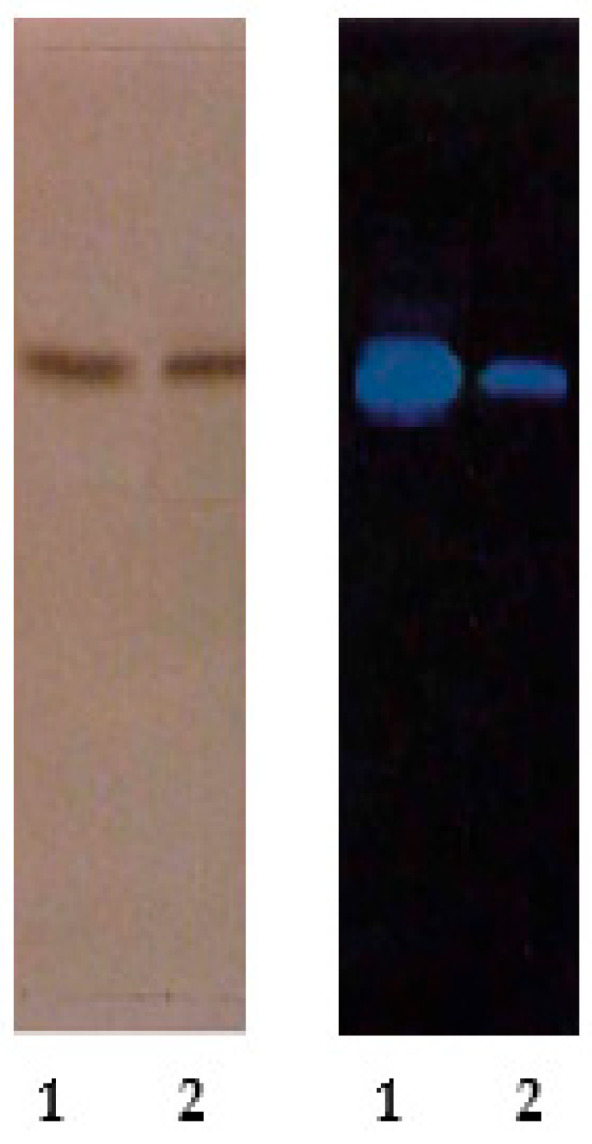
TLC profile of quercetin (isolate) (1); quercetin (standard) (2). Visualization was performed citroborate reagent under UV light at 254 nm and under UV light at 254 nm.

Figure 3 shows TLC profile of quercetin (isolate) and quercetin (standard). A single band in TLC, characterized by UV, FTIR, NMR spectroscopy, and mass spectroscopy, was identified as quercetin. The results of this study indicate that the ethyl acetate fraction of andaliman (*Zanthoxylum acanthopodium* DC.) fruit contains an effective potential compound. Quercetin isolate is a yellow crystal, with m.p. 315–316 °C. The empirical formula was C_15_H_10_O_7_.

##### UV-Vis Spectrum

Based on the UV-Vis spectral data, the compound exhibited maximum absorbance at wavelengths of 369.5 nm. The maximum absorbance at 369.5 nm indicates the presence of quercetin’s characteristic conjugated aromatic system, confirming its identity and providing a basis for quantitative analysis.

##### FTIR Spectrum

Based on the FTIR spectral data, the compound contains functional groups such as C-O aromatic ether (1022.00); C=C aromatic (1452.62 and 1418.17); C=O aromatic ketone (1653.57); CH aromatic (2945.42 and 2830.59); and OH aromatic broadened (3324.37).

The FTIR spectrum supports the molecular structure of quercetin, confirming the presence of hydroxyls, aromatic rings, ketone groups, and ether linkages, which are essential features of its chemical identity.

##### Mass Spectrum

**Figure 4:**
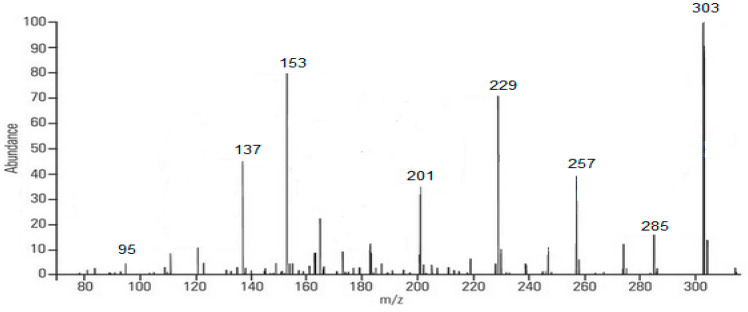
Fragmentation (MS/MS) spectrum of quercetin (*m*/*z* 303).

Figure 4 shows the fragmentation (MS/MS) spectrum of quercetin (*m*/*z* 303).

**Scheme 1:**
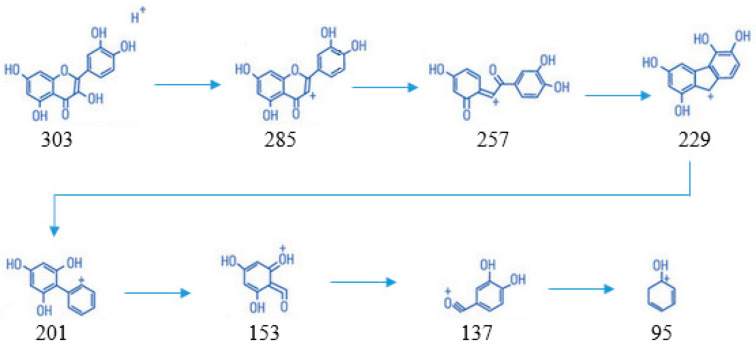
The compound structure of each ion from fragmentation of quercetin.

Based on Figure 4, the mass spectrum results obtained in negative mode showed the emergence of ions *m*/*z*, namely 303, followed by its fragmentation, including 285; 257; 229; 201; 153; 137; and 95. The compound structure of each ion can be seen below (Scheme 1).

##### NMR Spectrum

**Figure 5:**
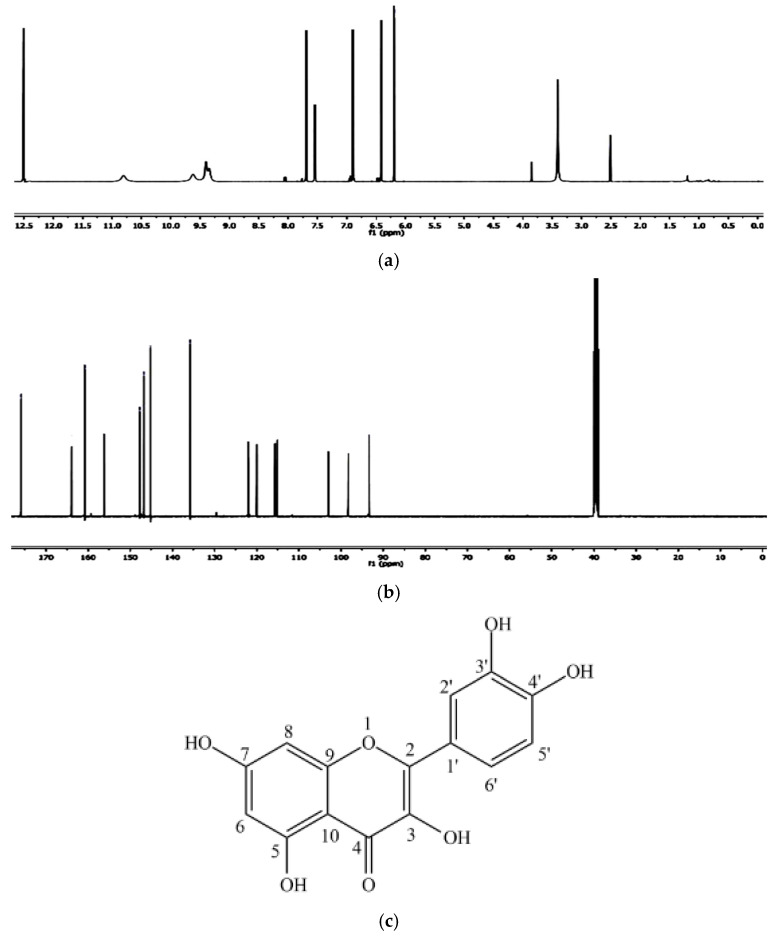
(**a**) ^1^H NMR spectrum of the isolated compound quercetin from andaliman fruit, (**b**) ^13^C NMR spectrum of the isolated compound quercetin from andaliman fruit, and (**c**) quercetin chemical structure.

Figure 5a,b shows the ^1^H NMR spectrum and ^13^C NMR spectrum of the isolated compound quercetin from andaliman fruit.

In the ^1^H-NMR spectrum, a combination of nine peaks was observed at chemical shifts of 6.22; 6.43; 6.87; 7.53; 7.67; 9.38; 9.67; 10.91; and 12.51 ppm. The isolated compound ^1^H-NMR spectrum showed aromatic hydrogen groups from 6.22–7.67 ppm and phenolic-OH groups from 9.38–12.51 ppm. The two bifurcated peaks, each with an integral of 1, a 2.2 Hz J-coupling, and chemical shifts of 6.43 and 6.22 ppm, belong to the aromatic ring A hydrogens, each split by a proton in its ortho position. The bifurcation peak with an integral of 1, chemical displacement of 6.87 ppm, and fission of 8.6 Hz are related to the proton of the aromatic B ring. The bifurcation peak with an integral of 1, chemical displacement of 7.67 ppm, and fission of 2.3 Hz are associated with the other hydrogen in the aromatic B ring. The singlet peak in the 12.51 ppm chemical displacement corresponds to the OH group (hydroxy) on carbon No. 5 in the A ring, which forms an intramolecular hydrogen bond with the carbonyl group.

From the ^13^C-NMR spectrum, this compound also has 15 carbons, and their chemical shifts are shown in Table 1. The peak with the highest chemical shift at 176.11 ppm belongs to the carbonyl group in the structure. Carbon peaks for aromatic groups were shown in the chemical shift of 94.12–164.85 ppm. The corresponding ^1^H-NMR and ^13^C-NMR showed the position of peaks for isolated compounds (Table 4). The peaks in the NMR spectrum showed a resemblance with pure quercetin, which was also confirmed by previous literature. Thus, this can ensure that the isolated compound is quercetin. Figure 5c shows a quercetin chemical structure.

The results of the spectroscopic analyses—comprising UV-Vis, FT-IR, NMR, and mass spectrometry—confirmed that the compound isolated from andaliman (*Zanthoxylum acanthopodium* DC.) fruit as an immunomodulator is a flavonoid belonging to the flavonol subclass. The compound was identified as 3,3′,4′,5,7-pentahydroxyflavone, commonly known as quercetin, with the molecular formula C_15_H_10_O_7_. The molecular structure of quercetin is presented in Figure 5c.

#### 2.1.6. Analysis of Compound Content Using LC-HRMS

The results obtained from the UHPLC–HRMS instrument are in the form of a chromatogram. Each chromatogram peak indicates the presence of one compound. The chromatogram is then processed using the Compound Discovery 3 application so that the *m*/*z* spectra can be identified; thus, the molecular formula of the interpreted compound can be predicted. The predicted molecular formula is then used to search for the compound’s name with the aid of the mzcloud database and the website, https://www.chemspider.com/ (accessed on 1 August 2023). After obtaining the name of the compound and its structure through the mzcloud database and website, the measured *m*/*z* is compared with the calculated *m*/*z* by drawing the structure of the compound in question in the ChempDraw application.

**Figure 6:**
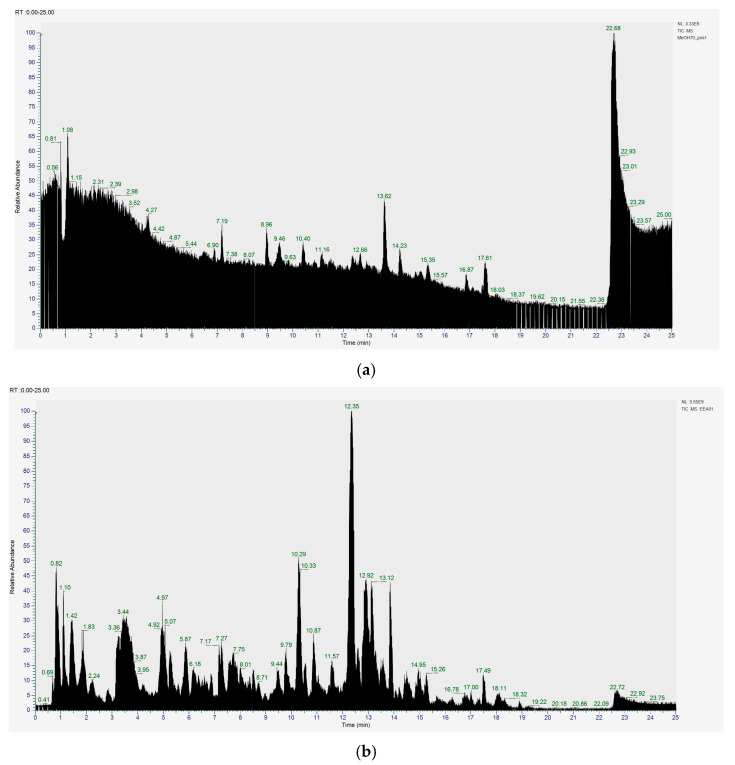
Total ion chromatogram (**a**) blank and (**b**) ethanol extract of andaliman (*Zanthoxylum acanthopodium* DC.) fruit.

Figure 6a shows the total ion chromatogram of the blank and the total ion chromatogram of the ethanol extract of andaliman (*Zanthoxylum acanthopodium* DC.) fruit. Based on the chromatogram interpretation for each peak, the predicted data for the andaliman (*Zanthoxylum acanthopodium* DC.) fruit compound identified in mzCloud Best Match showed match scores above 90%; 187 compounds were obtained, and based on the literature, 22 compounds have potential to act as immunomodulatory agents, as shown in Table 5. Eleven compounds that have potential to act as immunomodulatory agents are (2E,4E)-N-(2-methyl propyl) dodeca-2,4-dienamide, trans-3-Indoleacrylic acid, quercetin, ethylmorphine, α-Linolenic acid, L-Isoleucine, D-(+)-Pipecolinic acid, acetylcholine, kaempferol, α-Eleostearic acid, and isorhamnetin.

Based on the results obtained regarding the compound profile contained in the andaliman ethanol extract, it was stated that this sample contained the most flavonoids and phenolic compounds, followed by fatty acids and alkaloids, and several contaminant compounds were from the phthalate group.

Figure 6b shows the UHPLC–HRMS spectrum of ethanol extract of andaliman (*Zanthoxylum acanthopodium* DC.) fruit that detected several compounds, including the following: 1-Dodecyl-2-pyrrolidinone; 6-Methylquinoline; N-Methyltryptamine; bufotenin; 3-[(2-phenyl-1H-imidazol-4-yl) methylene]-1,3-dihydro-2H-indol-2-one; 4,7,8-trimethoxyfuro[2,3-b]quinolone; Bis(2-ethylhexyl) phthalate; oleamide; Bis(3,5,5-trimethyl hexyl) phthalate; ethylmorphine; D-(+)-Camphor; methyl isonicotinate; (2E,4E)-N-(2-methyl propyl) dodeca-2,4-dienamide; trans-3-Indoleacrylic acid; quercetin; ethylmorphine; α-Linolenic acid; L-Isoleucine; D-(+)-Pipecolinic acid; acetylcholine; kaempferol; α-Eleostearic acid; isorhamnetin; etc.

Predictions of molecular formulas and structures for 22 compounds contained in the ethanol extract that have potential to act as immunomodulatory agents can be seen in Table 5.

#### 2.1.7. Comparison of Chemical Compound Content in Andaliman Extract

**Figure 7:**
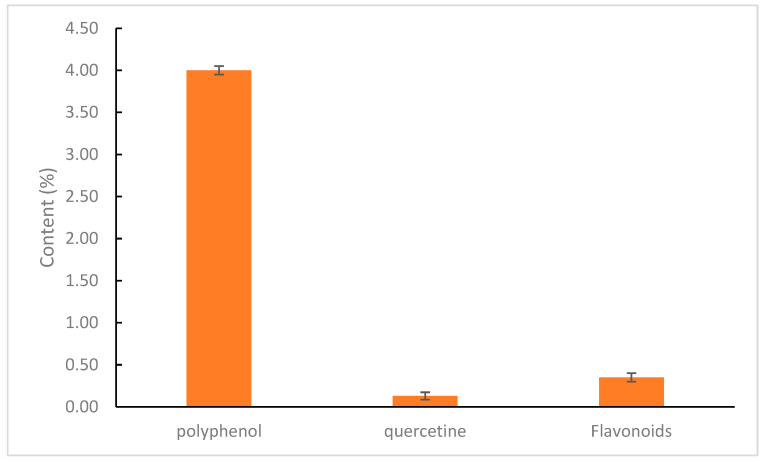
Chemical content of andaliman fruit extract.

A comparison of compound contents in the ethanol extract of andaliman fruit is illustrated in Figure 7.

**a.**  **Total Phenolic Content**

Quantitative identification of phenolic compounds in andaliman fruit extract was conducted to measure the total phenolic content, which plays a crucial role in contributing to antioxidant activity and is highly correlated to immunomodulator activity. Phenolic compounds help combat reactive oxygen species (ROS) and free radicals by inhibiting their initiation, disrupting chain reactions, and suppressing the formation of radicals such as singlet oxygen and hydrogen peroxide. One commonly used method for determining total phenolic content is the UV-Vis spectrophotometry method based on the colorimetric principle. This method requires a standard compound, typically gallic or caffeic acids, to determine the extract’s total concentration of phenolic hydroxyl groups. The Folin–Ciocalteu reagent (H_3_PO_3_(MoO_3_)_12_) at a concentration of 7.5% in water is used as a redox reagent. It reacts specifically with phenolic compounds in the extract to form a blue complex, which can be quantified using UV-Vis spectrophotometry. This occurs because molybdenum metal (Mo(VI)) is reduced by antioxidant electron donors. The addition of 1% NaOH is necessary to create an alkaline environment, enabling the Folin–Ciocalteu reagent to react and induce the dissociation of phenolic compounds into phenolate ions. Before measurement, the solution was incubated at room temperature to allow the chemical reaction to complete [33]. A calibration curve was established using a series of gallic acid concentrations to obtain a linear regression equation. This allowed the total phenolic content (TPC) in the andaliman fruit extract to be determined. Based on the results, the total phenolic content of the extract was found to be 4%, with a regression value (R^2^) of 0.9992.

**b.**  **Total Flavonoid Content**

Quantitative identification of flavonoids in andaliman fruit extract was performed to measure total flavonoid content, contributing to its antioxidant and immunomodulatory potential. Adding aluminum chloride to the standard solution triggers a reaction with flavonoid compounds, forming a red solution, the absorbance of which is then measured. The principle behind determining flavonoid levels with aluminum chloride is based on observing the formation of a complex between the keto group at the C-4 atom and the hydroxy group at the adjacent C-3 or C-5 atoms in flavone and flavonol groups.

Quercetin is used as a marker because it is a flavonoid from the flavonol group, which possesses a keto group at the C-4 atom and a hydroxy group at the adjacent C-3 or C-5 atoms. This allows the formation of an orange-red complex. The solution was then supplemented with ethanol, distilled water, and sodium acetate [34,35]. A calibration curve was created by preparing a series of concentrations, allowing the determination of a linear regression equation for total flavonoid content (TFC) in andaliman fruit extract. Based on the research, the regression equation obtained was y = 0.019x + 0.115, with a coefficient of determination (R2) of 0.997. The R2 value approaching 1 indicates a strong linear relationship between the concentration of the quercetin solution and its absorbance value. Based on these results, the total flavonoid content in andaliman fruit extract was determined to be 0.35%.

**c.**  **Quercetin Level**

Quantitative identification of quercetin in andaliman fruit extract was conducted to measure its total concentration, as quercetin plays a vital role in contributing to antioxidant and immunomodulator activity by combating reactive oxygen species (ROS) and free radicals. It does so by inhibiting the initiation of free radicals, breaking chain reactions, and suppressing the formation of free radicals such as singlet oxygen and hydrogen peroxide. Quercetin concentration was determined using the high-performance liquid chromatography (HPLC) method based on chromatography principles. This method requires a quercetin standard to determine the concentration of quercetin in the extract. Measurements were conducted at 360 nm—the optimal wavelength for quercetin detection in HPLC. A calibration curve was established to determine quercetin concentration in andaliman fruit extract by preparing a series of quercetin concentrations and generating a linear regression equation. The results showed that the total quercetin content in andaliman fruit extract was 0.13%, with a regression value (R2) of 0.9847.

#### 2.1.8. Development of Andaliman Extract Soft Capsule

**a.**  **Evaluation of Soft Capsule Shell**

An incompatibility study between the soft capsule shell containing andaliman extract and the excipients used in the soft capsule formulation showed that the soft capsule material was incompatible with propylenglycol and formed a soft, melted capsule shell (Table 6).

Next, the formulation stage involved developing the extract emulsion, which was performed without the use of propylene glycol as a solubilizer. The formulation was continued by the stress test. Further soft capsule formulations used formula A shells due to their good compatibility and minimal ingredients, making them the most efficient.

**b.**  **Formulation of Extract Emulsion**

In order to be formulated as soft capsules, andaliman extract was made into an emulsion consisting of an oil phase containing a solubilizer and a water phase containing extracts and emulsifiers. As a carrier in this study, the oil phase used variations of three oil types: soybean oil, sunflower oil, and medium chain triglycerides (MCT) oil (Table 7). Three types of surfactants were used as solubilizers in this phase: Montanox 20 (Polysorbat-20) with a concentration of 20 and 30%, and Gellucire 44/14 (Lauroyl Polyoxyl-32 glycerides), which is also a surfactant. The water phase of the emulsion contained andaliman fruit extract, which was made into an emulsion using two types of emulsifying agents: PEG 400 and Tween 80. These two phases were mixed with high-speed stirring to obtain a stable emulsion. The emulsion mixtures were then observed for stability in stress stability testing, and the results are shown in Table 8. The stress test was performed by centrifugation 500 and 1000 rpm speeds for 5 min each. In addition to the stress test, the compatibility test of the andaliman emulsion with the capsule shell was also conducted by storing the mixture in a climatic chamber.

Table 8 shows the characteristics of the emulsion extract of andaliman. The stress test results showed that among 18 formula combinations tested, two formulations were stable and homogeneous: F3.3E and F3.3F. These formulas were emulsions containing a 30% MCT oil phase and 5% Gellucire 44/14 (Lauroyl Polyoxyl-32 glycerides or lauroyl PEG-32 glycerides) as a solubilizer, mixed with andaliman extract in the water phase. F3.3E used PEG 400 (35%) as the emulsifier in the water phase, while the second formula (F3.3F) used the same oil phase but with 35% Tween 80 as the emulsifier. In general, it can be concluded that a stable emulsion is one that uses an MCT oil phase, which contributes to stability due to its shorter C chain length (6–12) compared to the soybean and sunflower oil used in the other two formulas. Shorter chains lead to easier emulsification. Further stress tests to identify the best emulsion formula were performed by centrifugation at 500 rpm and 1000 rpm for 5 min each. The results showed that the andaliman emulsion formula that remained homogeneous and stable was F3.3F. This formula used polysorbate 80 as an emulsifier, which showed stronger emulsification ability than polyethylenglycol (PEG) in F3.3E. The stable formula (F3.3F) was selected for further development, including pilot-scale encapsulation. F3.3F contains a 30% MCT oil phase with lauroyl PEG-32 glycerides as a solubilizer, and a water phase containing 35% polysorbate (Tween) 80 as the emulsifier. Tween 80, a non-ionic surfactant (polyoxytilene (20) sorbitan monooleate), is more susceptible during physical stability testing, namely centrifugation, due to its excellent wetting and washing functions, strong detergency, and superior emulsifying properties compared to the PEG emulsifier used in F3.3E. Tween has the advantages of good salt resistance and pH tolerance and is very effective at solubilizing oils and other hydrophobic substances.

**c.**  **Encapsulation of Andaliman Extract Soft Capsules**

**Figure 8:**
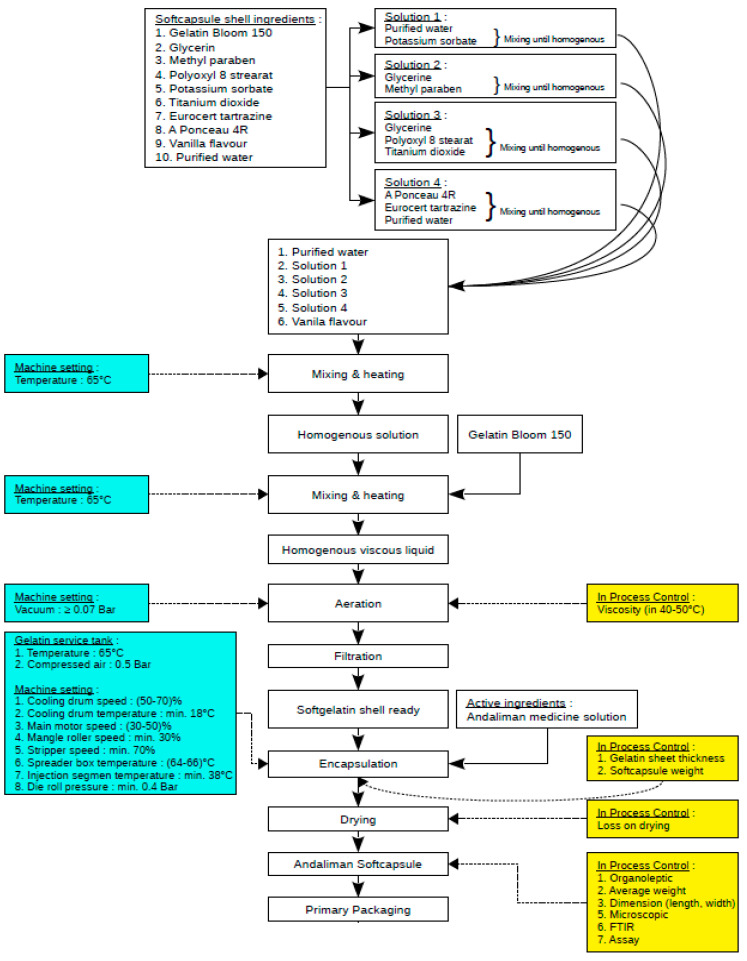
Process diagram and scale-up process parameters for andaliman emulsion encapsulation.

**Figure 9:**
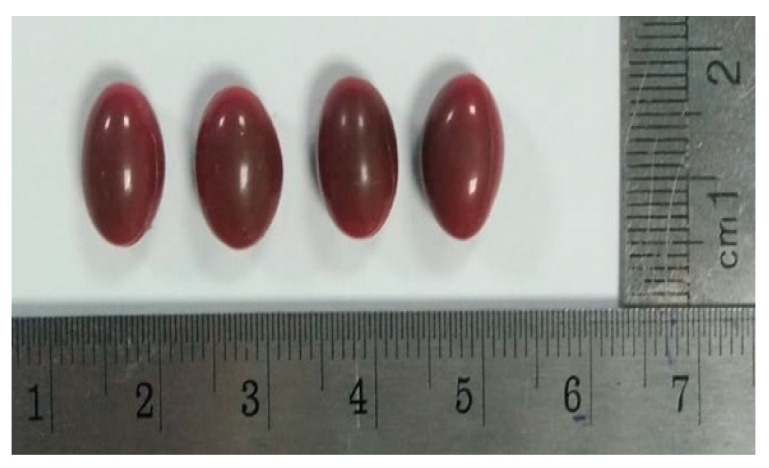
Andaliman extract soft capsule product.

The following process involves the encapsulation of the andaliman emulsion using five oval molds with filling volumes ranging from 0.265–0.308 mL. The encapsulation process is conducted at a gelatin temperature of 65 °C and compressed to 0.5 bar to maintain the gelatin in the form of a viscous, bubble-free solution. The pilot-scale encapsulation process of the andaliman emulsion can be seen in Figure 8. Soft capsule products (Figure 9) were then tested with characterization parameters based on BPOM requirements regarding the quality and safety of traditional medicines, with the results shown in Table 9.

#### 2.1.9. Immunomodulatory Activity Test on Lymphocyte Cells

Immune function can be evaluated by measuring the viability of lymphocytes. Cell viability testing serves as a preliminary assessment of potential immunomodulatory activity. This test offers several advantages, such as being cost-effective and relatively time-efficient and utilizing cells derived from humans, making it more relevant to the expected research outcomes [36]. Lymphocyte cells were isolated from the spleen of mice, as it is the primary secondary lymphoid organ containing T cells and B cells [1]. Testing was performed on a 96-well microplate with approximately 1300 cells per well. This cell density was selected to ensure that immune cells could complete their life cycle adequately within a 24 h incubation period. The 24 h incubation time was chosen to prevent a decrease in the availability of nutrients consumed by the cells. Andaliman ethanol extract was tested at 15.625; 31.25; and 62.5 ppm concentrations, with controls added to the wells. After a 24 h incubation, 10 μL of WST-8 reagent was added to each well, and the plate was incubated for an additional 2 h. This allowed the tetrazolium salt to react and form formazan salt, producing an orange color. Absorbance measurements were taken using a microplate reader at a wavelength of 450 nm. The higher the absorbance intensity of the formazan salt, or the more intense the orange color observed, the greater the number of living cells [37]. The parameter used in this test was the percentage of viability. The viability percentage represents the proportion of living cells at the end of the treatment relative to the initial number of cells at the start of the experiment.

Similarly, immune function can be evaluated through macrophage phagocytosis, an immunological parameter. This method is one of the most widely used techniques for screening active ingredients that influence immunity. Phagocytosis activity can be assessed by measuring the phagocytosis index and phagocytosis capacity [38]. Lymphocyte proliferation describes the response of lymphocytes to antigenic stimuli. Lymphocytes recognize and respond to foreign antigens and can mediate humoral and cellular immunity. The effects of extracts on lymphocyte proliferation can be quantified by measuring the proliferation stimulation index [16].

Cell culture isolates cells from tissues or organs and grows them under aseptic conditions using cell media that support in vitro growth. Cells can be isolated directly from tissues and then selected either enzymatically or mechanically before being cultured. Alternatively, they can be derived from cell line cultures or cell strains. The advantage of in vitro cell culture is the ability to control the environment, allowing for relatively constant physiological conditions. However, the disadvantage of this technique is that it requires precise handling and preparation to create a viable model. Therefore, a thorough understanding of cell characteristics and phenotypes is essential for interpreting the results accurately.

Immunomodulatory activity was determined against lymphocyte cells by culturing and incubating the cells for 24 h. RPMI 1640 is an effective medium for lymphocyte cell culture because it contains organic salts, amino acids, vitamins, and glucose. However, prolonged use of glucose in lymphocyte cells for more than 48 h can result in suboptimal cell proliferation [39]. Additionally, the RPMI culture medium was supplemented with fetal bovine serum (FBS) as a source of nutrients, hormones, and growth factors [40]. L-glutamine is a nutritional component for cell metabolism, and penicillin-streptomycin is an antimicrobial agent to prevent bacterial contamination during cell growth. Cell culture was maintained at 37 °C, consistent with human body temperature. Furthermore, CO_2_ was provided to ensure proper cell reproduction and growth [40].

Cell viability was assessed using the water-soluble tetrazolium (WST-8) method. This method is based on reducing WST tetrazolium salt to formazan by the activity of the dehydrogenase enzyme in cells, resulting in an orange color from the formazan dissolved in the culture medium (Dojindo). WST-8 is preferred because it is more stable and has lower cytotoxicity than the WST-1 and MTT assays. It also offers a more straightforward testing procedure and eliminates the need for additional steps to dissolve the formazan, as it produces water-soluble formazan during cellular reduction. Increased cell viability indicates that the extract is non-toxic to immune cells and has the potential to modulate cellular immune responses [41].

**Figure 10:**
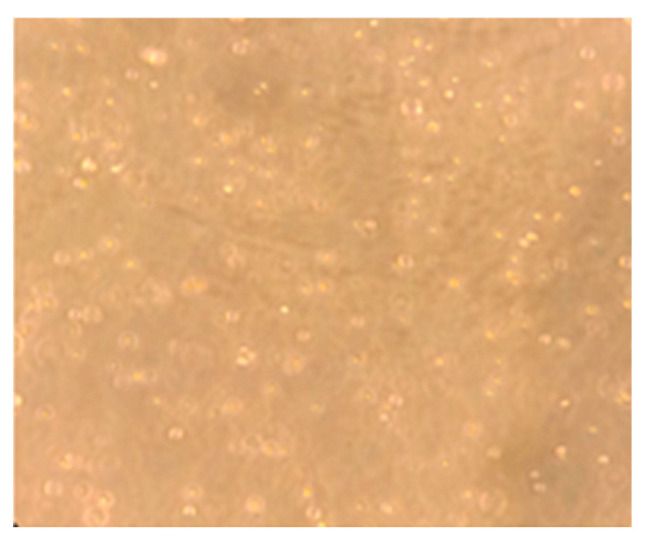
Lymphocyte cells cultured in RPMI 1640 medium.

**Figure 11:**
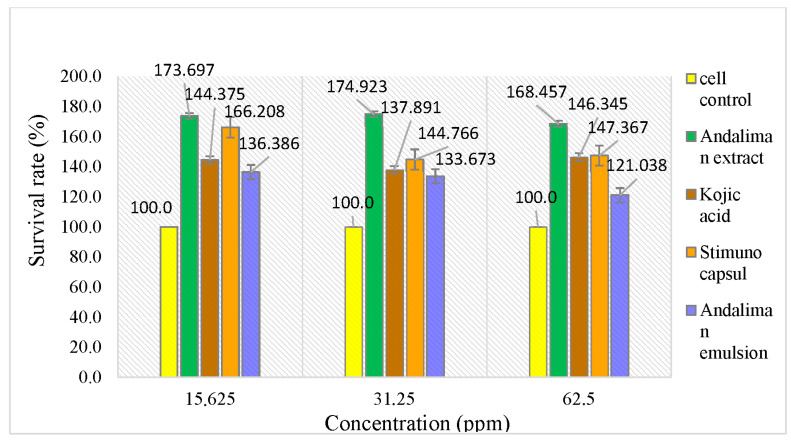
Viability of lymphocyte cells in andaliman ethanol extract, comparing andaliman extract and kojic acid.

Lymphocyte cells cultured in RPMI 1640 medium are shown in Figure 10. The effect of increasing andaliman fruit ethanol extract concentrations on cell viability was assessed using the WST-8 method, as presented in Table 10. The viability of lymphocyte cells treated with andaliman ethanol extract and kojic acid is illustrated in Figure 11.

Kojic acid was used as a positive control for comparison because it is known to influence the innate immune response in vitro using lymphocyte cells. Kojic acid promotes the differentiation of monocytes into macrophages, thereby acting as an immunomodulatory agent [42]. The test results show that kojic acid affects lymphocyte cell viability. The survival rate (%) of lymphocyte cells treated with andaliman ethanol extract was compared to that of kojic acid. The results indicate that the viability of lymphocyte cells in tests with solutions above 100% suggests that all test solutions can maintain cell viability. Additionally, viability results were analyzed using the one-way ANOVA method to assess differences between test groups and concentrations within each test group.

## 3. Materials and Methods

### 3.1. Materials

Andaliman (Zanthoxylum acanthopodium DC.) fruit was obtained from Medan, Indonesia. Afterwards, 400 g of andaliman fruit simplicia were macerated in 3 L of 70% ethanol. The samples were immersed for 3 × 24 h, with occasional stirring. The filtrate was concentrated using a vacuum rotary evaporator at 60 °C, 75 rpm. Further evaporation was performed using a water bath at 60 °C [43].

### 3.2. Methods

#### 3.2.1. Pharmacognosy Study

a. FTIR-ATR Testing

The FTIR instrument was first validated for wavelength accuracy using polystyrene film. It was then set to ATR mode, and the powdered andaliman fruit sample was placed on the crystal surface. The sample was pressed against the crystal using an FTIR press tool until a pressure of 80 gauss was achieved [19,20,44,45,46,47].

b. Extract Parameter Testing

Extract parameter testing included specific and nonspecific evaluations. Specific parameters tested were organoleptic characteristics, macroscopic and microscopic observations, chromatographic profiles, and chemical compound content. Nonspecific parameters included moisture content (loss on drying), specific gravity, water content, total ash, acid-insoluble ash, microbial contamination, and heavy metal contamination [4].

c. Phytochemical Screening

Phytochemical screening was conducted as a preliminary qualitative analysis to identify the presence of major chemical constituents. The screening included tests for alkaloids, flavonoids, polyphenols, quinones, monoterpenoids, terpenoids, steroids, saponins, and tannins, using specific colorimetric reagents or precipitation methods [48,49].

d. Thin Layer Chromatography (TLC) Profile of the Extract

A 0.1% solution of andaliman fruit extract was prepared by dissolving 1 mg of extract in 1 mL of ethanol (p.a.) and sonicating it for 15 min using an ultrasonic cleaner. The mobile phase for TLC development consisted of ethyl acetate/n-hexane/formic acid (7:3:0.5); 0.1% quercetin solution was used as a reference compound. The extract and the quercetin reference were detected using a cytoroborate spot detector under a UV lamp of 366 nm.

e. Isolation of Quercetin

The ethanol extract of andaliman fruit (25 g) was fractionated by liquid–liquid extraction using eluents with increasing solvent polarity to give fractions. The solvents were n-hexane, ethyl acetate, and water in increasing polarities, respectively. Briefly, the ethyl acetate extract was purified to isolate quercetin by column chromatography using a combination of eluents.

Column chromatography was used to separate quercetin from the ethyl acetate fraction of andaliman fruit. This method used a column with a diameter of 1 cm and a height of 100 cm, filled with Sephadex LH-20 and 80% methanol as the solvent. Then, 300 mg of ethyl acetate fraction was dissolved with 80% methanol solvent and was loaded on the column. The mobile phase was 80% methanol. After adding 280 mL of 80% methanol, the first compounds were withdrawn from the column.

f. Structure Elucidation: Nuclear Magnetic Resonance (NMR) Analysis

Proton nuclear magnetic resonance (1H-NMR) and carbon nuclear magnetic resonance (13C-NMR) spectra were recorded on a Bruker 500 MHz AVANCE III HD NMR Spectrometer, using tetramethyl silane (TMS) as the internal standard. The solvents were hexadeuterodimethyl sulfoxide (DMSO-d6). All data compared with that of the literature and previous data.

#### 3.2.2. Analysis of Compound Content Using LC-HRMS

Examination was conducted by employing liquid chromatography with a Thermo Scientific™ Vanquish™ UHPLC Binary Pump and Orbitrap high-resolution mass spectrometry utilizing a Thermo Scientific™ Q Exactive™ Hybrid Quadrupole-Orbitrap™ High-Resolution Mass Spectrometer. The liquid chromatography procedure utilized a Thermo Scientific™ Accucore™ Phenyl-Hexyl analytical column with dimensions of 100 mm × 2.1 mm ID × 2.6 µm. The mobile phases consisted of MS-grade water with 0.1% formic acid (A) and MS-grade methanol with 0.1% formic acid (B), following a gradient technique at a 0.3 mL/min flow rate. Initially, mobile phase B was set at 5%, gradually increasing to 90% over 16 min, holding at 90% for 4 min, and then returning to the initial condition (5% B) by 25 min. The column temperature was maintained at 40 °C, and the injection volume was 3 µL. Untargeted screening was performed using full MS/dd-MS2 acquisition mode in positive or negative ionization polarities/states. Nitrogen served as sheath, auxiliary, and sweep gases at 32, 8, and 4 arbitrary units (AU), respectively. The spray voltage was 3.30 kV, the capillary temperature was set at 320 °C, and the auxiliary gas heater temperature was set at 30 °C. The scan range spanned 66.7–1000 *m*/*z*, with a resolution of 70,000 for full MS and 17,500 for dd-MS2 in positive ionization modes. The system was controlled by XCalibur 4.4 software from Thermo Scientific, Bremen, Germany.

#### 3.2.3. Analysis of Chemical Compound Content

a. Total Phenol Content

Total phenol content was determined using gallic acid as the standard. Approximately 0.2 mL of Folin–Ciocalteu reagent and 0.5 mL of sodium carbonate (Na_2_CO_3_) solution were added to the blank, sample, and standard solutions in separate vortex tubes. Subsequently, 8.8 mL of distilled water was added to each tube. The absorbance of each test solution was then measured using a spectrophotometer [50,51,52].

b. Total Flavonoid Content

Quercetin was used as the standard, prepared by dissolving it in ethanol p.a., followed by serial dilutions to obtain concentrations of 10, 20, 30, and 40 ppm. The extract was dissolved in 25 mL of ethanol p.a., and 0.1 mL of 10% aluminum chloride (AlCl_3_), 0.1 mL of 1 M sodium acetate (CH_3_COONa), and 2.8 mL of distilled water were added. The mixture was incubated in a closed container for 30 min at room temperature, and the absorbance was measured using a spectrophotometer.

c. Quercetin Levels

Quercetin content was analyzed at a detection wavelength of 725 nm using an HPLC system equipped with a SunFire C18 column (15 cm × 4.6 mm × 5 µm). The mobile phase consisted of methanol and 0.2% phosphoric acid (H_3_PO_4_) in a ratio of 65:35. Methanol was used as the solvent, with a flow rate of 1 mL/min, an injection volume of 10 µL, and detection at a wavelength of 360 nm. The retention time (RT) was approximately 6 min. A calibration curve was established to quantify the quercetin levels in the extract.

#### 3.2.4. Soft Capsule Development

##### Incompatibility Study of Extract with Capsule Shell 

The soft capsule shell was formulated using three variations, as shown in the following Table 11.

Each capsule shell formula was subjected to an incompatibility test to evaluate its interaction with the excipients used in the soft capsule emulsion. The test was conducted using a stress test method, which included centrifugation at 500 and 1000 rpm, each for 5 min. Subsequently, a stress stability test was performed by storing the samples in a climatic chamber.

#### 3.2.5. Immunomodulatory Activity Test on Lymphocyte Cells

A test solution of andaliman ethanolic extract and kojic acid (as the standard) was prepared as a stock solution with a concentration of 50,000 ppm in dimethyl sulfoxide (DMSO), ensuring that the DMSO concentration did not exceed 2%, as it is toxic to cells [51]. Subsequently, a serial dilution was performed to obtain concentrations of 2000, 1000, 500, 250, 125, 62.5, and 31.25 ppm. A solvent control was included to determine the potential cytotoxicity of the solvent on lymphocyte cells. This control contained only the culture medium, lymphocyte cells, and the solvent (DMSO) without any extract or standard compound.

Lymphocytes were isolated from the spleens of 10-week-old male Swiss Webster mice weighing approximately 25 g, which had been acclimatized for 7 days. RPMI 1640 medium (Roswell Park Memorial Institute) was perfused into the spleen to release the lymphocyte cells into the medium. Tris-ammonium chloride buffer was then added to exert minimal effects on leukocytes. Cell counting was performed by mixing the cell suspension with trypan blue dye and counting viable cells using a hemocytometer to determine the number of cells used in the viability assay.

##### Lymphocyte Cell Viability Test on Andaliman Extract

The viability test was conducted using a 96-well microplate, with approximately 1300 lymphocyte cells per well. Each well was treated with varying concentrations of andaliman extract at 15.625, 31.25, and 62.5 ppm, along with appropriate control groups. After an incubation period of 24 h, 10 µL of WST-8 reagent was added to each well. The plate was then incubated for another 2 h to allow the tetrazolium salt to react and form formazan salt, producing an orange color. Absorbance was measured using a microplate reader at a wavelength of 450 nm. A higher absorbance value, or a more intense orange color, indicates more viable (living) cells [37].

A higher percentage of cell viability corresponds to more remarkable cell survival. Cell viability was calculated using the following equation:

$$\%\;\mathrm{Cell}\;\mathrm{Viability}=\frac{A-B}{C-B}\;\times \;100\%$$

where

A = absorbance of the treatment group

B = absorbance of the media control

C = absorbance of the cell control

## 4. Conclusions

The fruit of *Z. acanthopodium*, which contains phenolic compounds known to modulate the immune response, was assessed by UHPLC-HRMS and FTIR. Eleven compounds have potential as immunomodulatory agents. The optimum formula, which contains a 30% MCT oil phase with lauroyl solubilizer PEG-32 glycerides and a water phase containing a 35% polysorbate (Tween) 80 emulsifier, was the best and most stable formulation. The immunomodulatory activity of the extract and its emulsion was assessed by measuring the viability of lymphocytes. Through extract profiling using UHPLC–HRMS and bioactivity testing, the study demonstrated that both the extract and its emulsion significantly enhanced lymphocyte viability, surpassing the standard immunomodulator kojic acid. This research illustrates the potential of andaliman extract as an immunomodulatory agent formulated as an emulsion in soft capsules.

## Figures and Tables

**Table 1:** Interpretation of functional groups in the FTIR-ATR profile [19,20].

No.	Detected Wavelength Range (cm^−1^)	Fresh Andaliman Fruit (cm^−1^)	Andaliman Simplicia (cm^−1^)	Andaliman Ethanol Extract (cm^−1^)	Identified Functional Group	Compound Group
1.	3500–3000	3278.95	3280.92	3344.63	O–H	Indication of polyphenols
2.	1800–1500	1630.41	1607.08	1563.01	C=O –C=C–	Indication of flavonoids and terpenoids
3.	1400–1000	1027.36 1422.84	1030.83 1416.59	1050.05 1405.58	C–O	Indication of aromatic groups in carbohydrates

**Table 2:** Characterization of Andaliman Fruit Extract.

No.	Parameters	Specification	Result of Ethanol Extract	Reference
**Specific Parameter**				
1.	Macroscopic characteristics (i.e., form, size, color, odor, and taste)			
(a)	Form	Thick solution	Thick solution	Internal Specification
(b)	Color	Brown to dark brown	Brown to dark brown	Internal Specification
(c)	pH (1% in aqueous solution)	5.0–7.0	6.5	Internal Specification
(d)	Total solid (%)	60–70	70	Internal Specification
(e)	Specific gravity (g/mL)	0.700–1.200	0.8100	Internal Specification
2.	Microbiological contamination			
(a)	Total plate count	≤10^4^ cfu/g	˂10^4^ cfu/g (90 cfu/g)	BPOM Regulation No. 32/2019 concerning Safety and Quality Requirements for Traditional Medicines
(b)	Total yeast and mold count	≤10^3^ cfu/g	˂10^3^ cfu/g (30 cfu/g)	
(c)	*Escherichia coli*	Negative/10 g	Negative	
(d)	*Salmonella* spp.	Negative/10 g	Negative	
**Nonspecific Parameter**				
1	Specific gravity	-	0.81 g/mL	Internal Specification
2	Drying loss (*w*/*w*)	-	13.6 ± 0.4%	Internal Specification
3	Water content	-	10.45 ± 0.02%	Internal Specification
4	Total ash content (*w*/*w*)	-	2.82 ± 0.07%	Internal Specification
5	Acid-insoluble ash content (*w*/*w*)	-	0.55 ± 0.13%	Internal Specification

**Table 3:** Metal contamination levels in andaliman fruit extract.

No.	Test Parameters	Level	Unit	Regulatory Limit
1	Lead (Pb)	≤0.001	mg/kg	≤10 mg/kg
2	Cadmium (Cd)	≤0.001	mg/kg	≤0.3 mg/kg
3	Mercury (Hg)	≤0.001	µg/kg	≤0.5 mg/kg

**Table 4:** ^1^H-NMR and ^13^C-NMR data for the isolated compound quercetin.

^1^H NMR Spectrum of Quercetin	^13^C NMR Spectrum of Quercetin
6.22 (d, 1H, *J* = 2.2 Hz, *Ar-H*)	94.12; 99.04; 116.01; 116.85; 120.24 (*Ar-C*)
6.43 (d, 1H, *J* = 2.2 Hz, *Ar-H*)	121.01; 123.14; 136.62 (*Ar-C*)
6.87 (d, 1H, *J* = 8.6 Hz, *Ar-H*)	146.02 (*Ar-C*)
7.53 (q, 1H, *J* = 8.5 Hz, *Ar-H*)	147.14 (*Ar-C*)
7.67 (d, 1H, *J* = 2.3 Hz, *Ar-H*)	148.35 (*Ar-C*)
9.38 (s, 2H, *Ar-OH*)	157.05 (*Ar-C*)
9.67 (s, 1H, *Ar-OH*)	161.54 (*Ar-C*)
10.91 (s, 1H, *Ar-OH*)	164.85 (*Ar-C*)
12.51 (s, 1H, *Ar-OH*)	176.11 (*Ar-C=O*)

**Table 5:** Predictions of active compounds in the ethanol extract of andaliman (*Zanthoxylum acanthopodium* DC.) fruit as immunomodulators.

No	Compound Name and Structure	Molecular Formula	Molecular Weight	Retention Time (min)	Area Max	Mz Cloud Best Match (%)	Concentration (%)	Activity Immunomodulator
1.	1-Dodecyl-2-pyrrolidinone 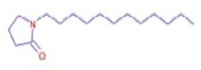	C16 H3 O	254.257	13.967	14,366,496,185.533	83	3.91	-
2.	6-Methylquinoline 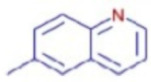	C10 H9 N	144.080	3.356	10,327,937,383.0783	97.4	3.48	-
3.	N-Methyltryptamine 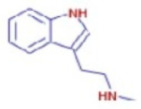	C11 H14 N2	175.122	3.36	5,861,404,729.80533	99.9	2.37	-
4	Bufotenin 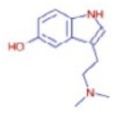	C12 H16 N2 O	2015.132	1.621	5,563,535,938.54227	99.8	1.52	-
5	3-[(2-phenyl-1H-imidazol-4-yl)methylene]-1,3-dihydro-2H-indol-2-one 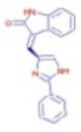	C18 H13 N3 O	288.122	10.977	4,543,172,895.765	98.3	1.421	-
6	(2E,4E)-N-(2-methylpropyl)dodeca-2,4-dienamide 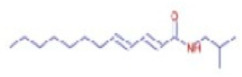	C16 H29 N O	252.232	13.402	3,934,736,043.8591	92.6	1.07	Immunomodulator
7	trans-3-Indoleacrylicacid 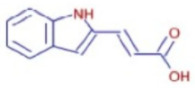	C11 H9 N O2	188.070	2.337	3,546,987,932.53118	89.4	0.97	Immunomodulator
8	4,7,8-trimethoxyfuro[2,3-b]quinoline 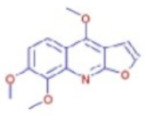	C14 H13 N O4	260.091	8.621	2,921,177,866.39903	99.3	0.80	-
9	Bis(2-ethylhexyl) Phthalate 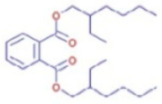	C24 H38 O4	391.283	17.603	2,720,019,234.75091	99.9	0.74	-
10	Oleamide 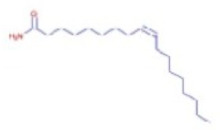	C18 H35 N O	282.278	15.039	2,444,410,257.87868	90.6	0.67	-
11	Bis(3,5,5-trimethylhexyl) phthalate 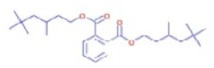	C26 H42 O4	419.314	18.203	2,107,105,993.16125	98.2	0.57	-
12	Quercetin 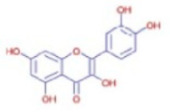	C15 H10 O7	303.049	7.698	2,106,441,300.66138	98.4	0.57	Immunomodulator
13	Ethylmorphine 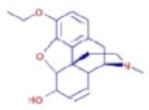	C19 H23 N O3	314.174	5.38	2,021,898,958.79495	82.5	0.55	-
14	D-(+)-Camphor 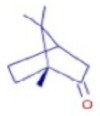	C10 H16 O	135.166	6.661	1,918,589,503.23951	84	0.52	-
15	Methyl isonicotinate 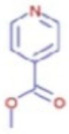	C7 H7 N O2	138.054	0.939	1,695,837,892.86535	97	0.46	-
16	α-Linolenic acid 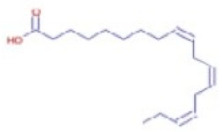	C18 H30 O2	279.231	14.638	1,185,635,190	99.7	0.34	Immunomodulator
17	L-Isoleucine 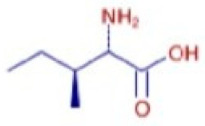	C6 H13 N O2	132,101	0.97	918,950,817.481148	99.9	0.26	Immunomodulator
18	D-(+)-Pipecolinic acid 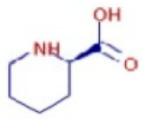	C6 H11 N O2	130.086	1.023	760,561,951.8	97.3	0.41	Immunomodulator
19	Acetylcholine 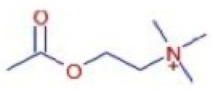	C7 H15 N O2	146.117	0.98	532,610,160.461447	81.7	0.15	Immunomodulator
20	Kaempferol 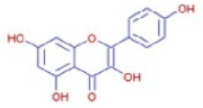	C15 H10 O6	287.054	8.62	199,010,654.590248	98.5	0.06	Immunomodulator
21	α-Eleostearic acid 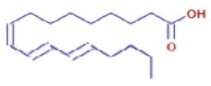	C18 H30 O2	279.231	12.723	517,667,258.026672	98.5	0.14	Immunomodulator
22	Isorhamnetin 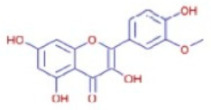	C16 H12 O7	317.064	8.808	490,460,053.433226	98.7	0.14	Immunomodulator

**Table 6:** Incompatibility study of the soft capsule shell containing other ingredients.

No.	Materials	Temperature (°C)	Shell Sheet A	Shell Sheet B	Shell Sheet C
(a)	Andaliman extract	30	(−)	(−)	(−)
		40	(−)	(−)	(−)
(b)	Propylenglycol	30	(+)	(+)	(+)
		40	(+)	(+)	(+)
(c)	Tween 80	30	(−)	(−)	(−)
		40	(−)	(−)	(−)
(d)	Gellucire 44/14	30	(−)	(−)	(−)
		40	(−)	(−)	(−)
(e)	PEG 400	30	(−)	(−)	(−)
		40	(−)	(−)	(−)
(f)	Montanox 20 ppi	30	(−)	(−)	(−)
		40	(−)	(−)	(−)
(g)	MCT Oil	30	(−)	(−)	(−)
		40	(−)	(−)	(−)
(h)	Sunflower Oil	30	(−)	(−)	(−)
		40	(−)	(−)	(−)
(i)	Soybean Oil	30	(−)	(-)	(−)
		40	(−)	(−)	(−)

Notes: (+) interaction found (soft, melted shell); (−) no interaction.

**Table 7:** Physical observation of the extract emulsion of andaliman.

	Water phase (Extract)		Andaliman extract (30%) using emulsifier:			
Oil phase (filler)						
			PEG 400 (20%) (A)		Tween 80 (20%) (B)	
SOYBEAN OIL 30% (F1)	Montanox 20 ppi (20%)	1	F1.1A	(−)	F1.1B	(−)
SUNFLOWER OIL 30% (F2)	Montanox 20 ppi (20%)		F2.1A	(−)	F2.1B	(−)
MCT OIL 30% (F3)	Montanox 20 ppi (20%)		F3.1A	(−)	F3.1B	(−)

	Water phase (Extract)		Andaliman extract (30%) using emulsifier:			
Oil phase (filler)						
			PEG 400 (10%) (C)		Tween 80 (10%) (D)	
SOYBEAN OIL 30% (F1)	Montanox 20 ppi (30%)	2	F1.2C	(−)	F1.2D	(−)
SUNFLOWER OIL 30% (F2)	Montanox 20 ppi (30%)		F2.2C	(−)	F2.2D	(−)
MCT OIL 30% (F3)	Montanox 20 ppi (30%)		F3.2C	(−)	F3.2D	(−)

	Water phase (Extract)		Andaliman extract (30%) using emulsifier:			
Oil phase (filler)						
			PEG 400 (35%) (E)		Tween 80 (35%) (F)	
SOYBEAN OIL 30% (F1)	Gellucire 44/14 (5%)	3	F1.3E	(−)	F1.3F	(−)
SUNFLOWER OIL 30% (F2)	Gellucire 44/14 (5%)		F2.3E	(−)	F2.3F	(−)
MCT OIL 30% (F3)	Gellucire 44/14 (5%)		**F3.3E**	**(+)**	**F3.3F**	**(+)**

Notes: (−) separation observed (creaming) (+) stable (homogeneous).

**Table 8:** Characteristics of the emulsion extract of andaliman.

Parameters	Results
Color, physical form	Brownish thick liquid
density (g/mL)	1.07 g/ml
Viscosity	927 cP
Microbial impurities	
- ALT (≤10^4^ cfu/g) *	<10 colony/g
- Yeast mold number (≤10^3^ cfu/g) *	<10 koloni/g
- *Eschericia coli*	Negatif
- *Salmonella* sp.	Negatif
Chemical content	
- Total polyphenol	1.53% ± 0.58
- Quercetine	0.03% ± 2.72

* Indonesian FDA Regulation No. 32, year 2019.

**Table 9:** Characterization of the andaliman extract soft capsule.

NO.	Test Parameter	Result
(a)	Appearance:	
	Shape	Soft Capsule
	Color	Maroon
	Size	Oval 5
	Length	11.50 mm
	Width	7.05 mm
(b)	Average individual weight	228–235 mg
(c)	Leakage	No Leakage
(d)	Disintegration time	15 min
(e)	Loss on drying	4.50%
(f)	Chemical compound content	
	- Polyphenol total	1.53%
	- Quercetin	0.03%
(g)	Microbiological contamination	
	Total plate count (spec: ≤10^5^ koloni/g)	7.7 × 10^2^
	Yeast and mold count (spec: ≤10^3^ koloni/g)	5.9 × 10^2^
	*E. coli* (spec: negative)	Negative
	*Enterobacteriaceae* (spec: ≤10^3^ koloni/g)	<10 koloni/g
	*Clostridia* (spec: negative)	Negative
	*Salmonella* sp. (spec: negative)	Negative
	*Staphylococcus aureus* (spec: negative)	Negative

**Table 10:** Survival rate (%) of lymphocyte cells after treatment with andaliman ethanol extract and kojic acid.

	Survival Rate (%)	
Concentration (ppm)	Andaliman Ethanol Extract	Kojic Acid
15.625	173.697 ± 0.003	144.375 ± 0.041
31.25	174.923 ± 0.013	137.891 ± 0.005
62.5	168.457 ± 0.021	146.345 ± 0.013

**Table 11:** Formulation of the soft capsule shell.

No.	Materials	Soft Capsule Shell Formula (in 50 g)					
		Formula A		Formula B		Formula C	
		Qty (g)	%	Qty (g)	%	Qty (g)	%
i	Gelatin	24.85	49.7	21.15	42.3	21.56	43.1
ii	Glycerin	6.31	12.6	5.94	11.9	0	0
iii	Sorbitol	0	0	4.86	9.7	9.53	19.1
iv	Purified water	18.84	37.7	18.05	36.1	18.91	37.8

## Data Availability

The original contributions presented in this study are included in the article. Further inquiries can be directed to the corresponding author(s).
